# The relationship between hours of sleep, screen time and frequency of food and drink consumption in Spain in the 2011 and 2013 ALADINO: a cross-sectional study

**DOI:** 10.1186/s12889-016-3962-4

**Published:** 2017-01-06

**Authors:** Napoleón Pérez-Farinós, Carmen Villar-Villalba, Ana María López Sobaler, María Ángeles Dal Re Saavedra, Aránzazu Aparicio, Sara Santos Sanz, Teresa Robledo de Dios, José Javier Castrodeza-Sanz, Rosa María Ortega Anta

**Affiliations:** 1 Spanish Agency for Consumer Affairs, Food Safety and Nutrition, Ministry of Health, Social Services and Equality, Alcalá 56, Madrid, 28014 Spain; 2 Department of Nutrition, Faculty of Pharmacy, Complutense University of Madrid, Plaza Ramón y Cajal s/n, Madrid, 28040 Spain

**Keywords:** Frequency of food consumption in children, Duration of sleep, Screen time, Educational level of parents

## Abstract

**Background:**

The frequency of intake of food and beverages depends on a number of ill-defined behaviour patterns. The objectives of this study were to evaluate the effects of screen time and sleep duration on food consumption frequency, and to describe frequencies and types of food consumption according to BMI category and parents’ level of education.

**Methods:**

We studied 6287 and 2806 children drawn from the 2011 and 2013 cross-sectional ALADINO studies respectively. Data were collected on number of hours of sleep, screen time, and weekly frequency of consumption of 17 food groups. Weight status was measured, and information was also collected on parents’ educational level. Average food consumption frequencies were calculated by reference to hours of sleep and hours of screen time, and were defined as ≥4 times or <4 times per week (once per week for soft drinks and diet soft drinks). Differences in frequency were evaluated for screen times of more and less than 2 h per day, and for sleep durations longer or shorter than the daily average. We fitted logistic regression models to evaluate the independent association between screen exposure and hours of sleep on the one hand, and food consumption frequency on the other.

**Results:**

Consumption of fruit and vegetables was lower among children who had parents with no formal or only primary school education. High levels of screen time were associated with a greater frequency of consumption of energy-dense, micronutrient-poor products and a lower frequency of consumption of fruit and vegetables. Sleeping a sufficient number of hours was associated with a higher consumption of fruit and vegetables. The results for 2011 were concordant with those for 2013.

**Conclusions:**

If efforts to ensure healthier eating habits among children are to be at all successful, they should focus on promoting a sufficient amount of sleep for children, limiting the time they spend watching television and/or playing with computers or video games, and educating parents accordingly.

## Background

Childhood overweight and obesity are an important public health problem in Europe. Prevalence remains high, though the trend seems to have reached a plateau [1].

Some of the factors linked to childhood obesity are lifestyle-related and, among others, include the daily amount of time which children devote to passive forms of entertainment, such as television, computers, mobile phones or video games, and which nowadays is very high. Relationships have been described between childhood obesity and the fact of having a television in the bedroom [2]. Parents’ educational level appears to be strongly associated with childhood obesity and lifestyle in terms of dietary habits and physical activity [3, 4].

The mechanism whereby screen time increases childhood obesity is unclear. In addition to the substitution of sedentary for active leisure activities [5, 6], it has been suggested that the cause may lie in excessive energy intake, since eating while watching television reduces the feeling of satiety [5, 7]. Another possibility considered is that exposure of children to advertisements for foods and drinks rich in saturated fats, sugar or salt heightens their desire to consume such products [8].

Furthermore, the use of televisions and computers appears to be associated with a reduction in the amount of sleep per day [9], and low levels of sleep are another factor known to be related to childhood obesity [10, 11]. Beyond the fact that sleeping less leaves children with more time for eating, the mechanisms whereby reduced sleep is linked to childhood obesity also remain unclear. Relationships have been suggested with endocrinological factors, which would involve cortisol, insulin, ghrelin and leptin. Hormonal changes may have an influence on appetite and food consumption, thereby promoting overweight and obesity through their dietary effects [12–15].

Accordingly, screen time and lack of sleep appear to be interrelated, affecting children’s food consumption in different ways. As these are modifiable habits, they are important when it comes to accounting for and -more particularly- preventing the increase in child obesity.

Hence, the aim of this study was: firstly, to evaluate the simultaneous effects of screen time and sleep duration on frequency of food consumption, adjusting for other variables; and secondly, to describe food consumption frequencies as a function of nutritional status and the parents’ level of education.

## Methods

### Setting and design

In 2006, the World Health Organisation (WHO) Regional Office for Europe launched the Childhood Obesity Surveillance Initiative (COSI), with the aim of using a common methodology to measure trends in obesity and overweight and their determinants among children aged 6–9 years, and so enable inter-country comparisons. Currently, the COSI has a membership of just under 30 countries.

Spain joined the COSI from its inception, through the Strategy for Nutrition, Physical Activity and Prevention of Obesity (*Estrategia para la Nutrición*, *Actividad Física*, *Prevención de la Obesidad y Salud*/*NAOS*) of the Spanish Agency for Consumer Affairs, Food Safety and Nutrition. The first data were collected in Spain during the 2^nd^ COSI round, in 2011. The resulting cross-sectional study was called the Nutrition, Physical Activity, Childhood Growth and Obesity (*Alimentación*, *Actividad Física*, *Desarrollo Infantil y Obesidad*/ALADINO) study in order to create an easily recognisable Spanish version of the COSI brand [16], and was repeated with the 3^rd^ COSI data-collection, resulting in the 2013 ALADINO Study.

After being discussed and approved by the participant countries, the main aspects of COSI implementation were embodied a general protocol which is in accordance with international ethical guidelines for biomedical research involving human subjects [17]. Subsequently, each country has made local arrangements in keeping with its national features and availability of resources.

The design and size of the samples, the age groups selected and other common methodological factors for COSI members are respectively based on protocols drawn up and published for this purpose [18]. This paper analyses the data collected in the 2011 and 2013 studies.

Following the sampling process, schools were contacted to seek their consent to taking part in the study. Where they responded in the affirmative, we contacted the parents of children in the classes selected, explained the features of the proposed study, and obtained their informed consent.

Three questionnaires were used, i.e., family (completed by parents), school (completed by school staff), and examiner. The examiner questionnaire included the anthropometric measurements of children’s weight, height and circumferences of waist and hip. The questionnaires were partially adapted to specific Spanish characteristics. Details of the questionnaires used have been previously published [19, 20].

### Sample

The 2011 ALADINO Study included 7638 boys and girls aged 6 to 9 years, and the 2013 study included 3666 boys and girls aged 7 to 8 years, from all parts of Spain.

The samples were representative of the Spanish population of these age ranges and sex. The samples for both studies were obtained using a multistage design. In the first stage, we established geographical strata based on the country’s Autonomous Regions. Within these strata, provinces were defined as clusters, with one or two provinces being selected by simple random sampling; similarly, within each province, municipalities were then selected by simple random sampling, depending on their population size; lastly, within each municipality, schools were selected, again by simple random sampling, taking into account whether they were publicly or privately owned.

For the purposes of this paper, we selected 6287 children (out of a total of 7638) in 2011, and 2806 children (out of a total of 3666) in 2013.

### Exposure variables

The exposure variables were sleep duration and screen time.

Sleep duration was reported by parents and recorded as hours and minutes of usual amount of sleep per day (including naps).

For multivariate analysis purposes, sleep duration was dichotomised into the categories, “short” (< population mean) and “long” (≥ population mean), based on the average sleep duration of the whole study population used for this analysis (9.9 h/day).

Information on screen time was assessed by ascertaining the number of hours per day the child usually spent watching TV (including videos), and/or playing computer (PC) or video games. The number of hours was ascertained separately for weekdays and weekends. Total screen time per day was computed as the sum of TV and PC hours, weighing weekdays (5/7) and weekends (2/7) as follows:

$$ \begin{array}{l}\mathrm{T}\mathrm{otal}\ \mathrm{s}\mathrm{creen}\ \mathrm{per}\ \mathrm{day}\ \left(\mathrm{hours}\right) = 5/7 \times \Big(\mathrm{reported}\ \mathrm{hours}\ \mathrm{of}\ \mathrm{P}\mathrm{C} + \mathrm{T}\mathrm{V}\ \mathrm{time}\ \mathrm{on}\ \\ {}\mathrm{weekdays}\Big) + 2/7 \times \left(\mathrm{reported}\ \mathrm{hours}\ \mathrm{of}\ \mathrm{P}\mathrm{C} + \mathrm{T}\mathrm{V}\ \mathrm{time}\ \mathrm{on}\ \mathrm{weekend}\ \mathrm{day}\mathrm{s}\right)\ \end{array} $$

For multivariate analysis, screen-time exposure was dichotomised into the categories, “low” (<2 h per day) and “high” (≥2 h per day), based on the recommendation of the American Academy of Paediatrics to limit total media time to no more than 1 to 2 h per day [21].

### Outcome variables

The outcome variables were frequencies of food and beverage consumption.

Information on the frequencies of the consumption of food and was obtained by means of the examiner’s form, which included a short food frequency questionnaire (FFQ) adapted from a Health Behaviour in School-aged Children study questionnaire that had been validated in adolescents [22]. The FFQ ascertained consumption frequencies over a typical week for several food items (in days per week), adapted to certain food products specific to Spain. The selected items were “legumes”, “100% fruit juice”, “whole-fat milk”, “milkshakes”, “cheese”, “yoghurt, cream cheese or other dairy products”, “meat”, “fish”, “potato chips and other salty snacks”, “candy bars or chocolate”, “biscuits and cakes”, “pizza, French fries (chips), hamburgers”, “eggs”, “cereals”, “white bread”, “whole-grain bread”, “pasta”, “fresh fruit”, “vegetables (excluding potatoes)”, “soft drinks containing sugar”, and “diet/light soft drinks”.

For the logistic regression models, the intake of each item was dichotomised into two categories, namely, “frequent consumption” (≥4 times per week) and “infrequent consumption” (<4 times per week). For the “soft drinks containing sugar” and “diet/light soft drinks” categories, “frequent consumption” was defined as “1 day or more per week” and “infrequent consumption” as “less than 1 day per week”.

### Covariates

The following covariates were selected: educational level of parents; nutritional status; outdoor playtime; and whether the child had a computer, television or games console in his/her bedroom.

Parents’ educational level was coded as one of three categories, “no formal or only primary school education”, “secondary school” or “university”, with the maximum educational level of one of the parents being taken.

The variable, children’s nutritional status, was estimated using Body Mass Index (BMI). Children’s weight and height were measured in accordance with WHO standardised techniques [23]. Children removed their shoes and socks, as well as any heavy clothing. Body weight was measured to the nearest 0.1 kg using portable digital (mainly manufacturer-calibrated) scales, and body height was measured to the nearest 0.1 cm using portable stadiometers, with subjects standing upright. BMI was calculated using the formula [weight (kg)]/[height (m)]^2^. To define overweight and obesity, we used the 2007 WHO recommended sex- and age-specific cut-offs for school-aged children and adolescents [24]. Overweight and obesity were defined as the proportion of children with a BMI value above +1 z-score and above +2 z-scores, respectively. Overweight included obesity.

The number of hours that children usually played outside (at home or somewhere else) was assessed for both weekdays and weekend days. As with the calculation of usual screen time, usual outdoor playtime was calculated by weighing weekday (5/7) and weekend hours (2/7) accordingly.

The presence of a passive form of entertainment in the bedroom was categorised as “yes” or “no”. Whereas these data had been collected in the 2011 survey using a single question, in 2013 they were collected using three questions, i.e., one for computers, one for televisions and another for video games. A single variable was subsequently obtained from the three questions, coded as “yes” when any of the three was answered in the affirmative.

### Statistical analysis

We performed a descriptive analysis of both the 2011 and 2013 samples, stratified by age, sex, educational level of parents and overweight (including obesity). Differences between children with and without overweight, according to sex and parents’ educational level, were assessed using the Chi-squared test.

The average times for sleep, exposure to screens and outdoor play, the proportion of children with a passive form of entertainment in their bedroom, and the frequency of consumption of each food item (days per week) were calculated for 2011 and 2013, according to weight status (normal weight or overweight) and maximum parental level of education (no formal or only primary school education; secondary school; university). We used the Student’s *t*-test for independent samples and analysis of variance (ANOVA) to test differences in means in the exposure and outcome variables, according to weight status and maximum parental educational level.

The average number of days of consumption of each food item was computed. Significant differences in consumption of food items, according to time of exposure to screens (<2 h or ≥2 h) and sleep time (above or below average), were assessed for both 2011 and 2013 using the Student’s *t*-test for independent samples.

Multivariable logistic regression models were fitted to assess the respective associations between screen time, sleep duration (exposure variables) and the frequencies of consumption of the 21 selected food items (outcome variables) in 2011 and 2013.

In the first phase, only screen time was included in the model. Sleep duration was subsequently added, to ascertain whether there was any change in the effects attributed to screen time. Once we had verified that such changes did not occur, both exposure variables were jointly included in the models.

Models were further adjusted for age (years), sex, outdoor playtime (hours/day), maximum parental educational level, BMI categories, and the presence of a computer, television or games console in the child’s bedroom.

## Results

A description of both 2011 and 2013 samples can be seen in Table 1: a total of 6287 children aged 6 to 9 years (49.6% girls, 50.4% boys) from the 2011 ALADINO Study and 2806 children aged 7 to 8 years (51.0% girls, 49.0% boys) from the 2013 ALADINO Study had data available on all variables. In both samples, the proportion of children with overweight was significantly lower among girls and children of parents with a higher educational level (Table 1).

**Table 1:** Study participants’ characteristics (total study group, both overall and by BMI category)

Characteristics		Total study group		Normal weight		Overweight		*p*-value
		n(%)		n(%)		n(%)		
2011								
Age								
	6	1585	24.4	972	61.3	613	38.7	
	7	1872	28.9	1029	55.0	843	45.0	
	8	1790	27.6	972	54.3	818	45.7	
	9	1240	19.1	658	53.1	582	46.9	
Sex								
	Boys	3269	50.4	1732	53.0	1537	47.0	
	Girls	3218	49.6	1899	59.0	1319	41.0	<0.001
Maximum parental level of education								
	Primary school	773	11.9	411	53.2	362	46.8	
	Secondary school	2512	38.7	1317	52.4	1195	47.6	
	University	3202	49.4	1903	59.4	1299	40.6	<0.001
		6487						
2013								
Age								
	7	1491	53.1	863	57.9	628	42.1	
	8	1315	46.9	724	55.1	591	44.9	
Sex								
	Boys	1374	49.0	745	54.2	629	45.8	
	Girls	1432	51.0	842	58.8	590	41.2	0.014
Maximum parental level of education								
	Primary school	259	9.2	129	49.8	130	50.2	
	Secondary school	1059	37.7	591	55.8	468	44.2	
	University	1488	53.0	867	58.3	621	41.7	0.033
		2806						

*p*-value for difference in proportions of overweight

Mean sleep duration was 9.9 h in both 2011 and 2013, with a mean of 9.8 for children with overweight and 9.9 for children with normal weight (*p* < 0.05) No statistically significant differences in sleep duration were found between boys and girls. (Table 2).

**Table 2:** Exposure variables, covariates and outcome variables for the total study group, both overall and stratified by body mass index category and country

Variables under study		Total study group		Normal weight		Overweight		Total study group		Normal weight		Overweight	
		2011						2013					
		Mean (SD)		Mean (SD)		Mean (SD)		Mean (SD)		Mean (SD)		Mean (SD)	
Sleep duration (hours/day)*		9.9	0.7	9.9	0.7	9.8	0.7	9.9	0.7	9.9	0.7	9.8	0.7
Screen time (hours/day)*		1.8	0.9	1.8	0.9	1.9	0.9	2.5	1.4	2.5	1.3	2.7	1.4
	Boys**	1.9	0.9	1.9	0.9	1.9	0.9	2.7	1.4	2.6	1.4	2.8	1.4
	Girls**	1.7	0.8	1.7	0.8	1.8	0.8	2.3	1.3	2.3	1.4	2.4	1.4
Outdoor play time (hours/day)		1.5	0.7	1.5	0.7	1.5	0.7	1.8	0.9	1.8	0.9	1.8	0.9
Have PC, TV, etc. in the room (%)*		30.2		26.7		34.7		35.1		32.6		38.4	
	Boys*	32.1		28.5		36.2		38.0		36.2		40.1	
	Girls*	28.3		25.1		32.9		32.3		29.4		36.5	
Outcome variables - food consumption frequencies (days/week)													
Legumes		2.6	1.4	2.5	1.4	2.6	1.5	2.6	1.4	2.6	1.4	2.6	1.4
100% fruit juice		2.9	2.5	2.8	2.5	3.0	2.5	2.4	2.3	2.3	2.3	2.5	2.3
Whole-fat milk*		3.8	3.3	4.1	3.2	3.4	3.3	3.4	3.3	3.8	3.3	2.8	3.2
Milkshakes		1.9	2.2	1.9	2.2	1.9	2.2	1.9	2.1	1.9	2.1	1.8	2.1
Cheese		3.0	2.2	3.0	2.2	3.1	2.2	2.9	2.1	2.9	2.1	2.8	2.1
Yoghourt, or other dairy products		4.7	2.2	4.7	2.2	4.7	2.2	4.6	2.2	4.6	2.2	4.7	2.2
Meat		3.7	1.9	3.7	1.8	3.6	1.9	3.6	1.9	3.7	1.9	3.6	1.8
Fish		2.7	1.5	2.7	1.5	2.6	1.5	2.7	1.5	2.7	1.5	2.7	1.5
Potato chips and other salty snacks		1.8	1.2	1.8	1.2	1.8	1.3	1.8	1.1	1.8	1.1	1.8	1.1
Candy bars or chocolate		2.0	1.5	2.1	1.5	2.0	1.5	2.1	1.4	2.1	1.5	2.0	1.3
Biscuits, cakes, etc.		2.4	1.8	2.5	1.8	2.4	1.7	2.7	2.0	2.9	2.0	2.6	1.9
Pizza, chips, hamburgers		2.0	1.0	2.0	1.0	2.0	1.0	2.0	0.9	2.0	0.9	2.0	0.9
Eggs		2.3	1.3	2.3	1.3	2.3	1.3	2.3	1.1	2.3	1.2	2.3	1.1
Cereals		2.9	2.5	3.0	2.5	2.8	2.4	2.7	2.4	2.8	2.5	2.6	2.4
White bread		4.6	2.6	4.7	2.5	4.5	2.6	4.5	2.5	4.6	2.5	4.4	2.5
Whole-grain bread		0.9	1.8	0.8	1.7	1.0	1.9	0.9	1.8	0.8	1.7	0.9	1.8
Pasta		2.7	1.6	2.8	1.7	2.7	1.6	2.5	1.4	2.6	1.5	2.4	1.2
Fresh fruit		4.8	2.3	4.8	2.3	4.8	2.4	4.9	2.3	5.0	2.3	4.9	2.3
Vegetables (excluding potatoes)		3.5	2.1	3.5	2.1	3.4	2.0	3.7	2.1	3.7	2.1	3.7	2.2
Soft drinks containing sugar		1.3	1.8	1.3	1.8	1.3	1.8	1.0	1.5	1.0	1.6	1.0	1.4
Diet/light soft drinks		0.7	1.7	0.6	1.5	0.8	1.8	0.5	1.3	0.5	1.2	0.6	1.4

* *p* < 0.05 for mean differences between weight status groups

** *p* < 0.05 for mean differences between weight status groups in 2013

Mean screen time was 1.8 h per day in 2011 (1.9 in boys and 1.7 in girls) and 2.5 h per day in 2013 (2.7 in boys and 2.3 in girls). In 2011, mean screen time was 1.9 h per day (1.9 in boys and 1.8 in girls) among children with overweight or obesity, and 1.8 h per day (1.9 in boys and 1.7 in girls) (*p* < 0.05) among children with normal weight, with the differences between boys and girls proving statistically significant. In 2013, mean screen time was 2.7 h per day (2.8 in boys and 2.4 in girls) among children with overweight or obesity, and 2.5 h per day (2.6 in boys and 2.3 in girls) (*p* < 0.05) among children with normal weight. Again, the differences between boys and girls were statistically significant (Table 2). Mean screen time was significantly higher among children (boys and girls) who had parents with no formal or only primary school education, in both 2011 and 2013 (Table 3).

**Table 3:** Exposure variables, covariates and outcome variables for the total study group, both overall and stratified by parents’ level of education

Variables under study	No formal or only primary school education		Secondary		University		No formal or only primary school education		Secondary		University	
	2011						2013					
	Mean (SD)		Mean (SD)		Mean (SD)		Mean (SD)		Mean (SD)		Mean (SD)	
Sleep duration (hours/day)*	9.8	0.8	9.9	0.7	9.9	0.7	9.8	0.8	9.8	0.8	9.9	0.6
Screen time (hours/day)*	2.0	0.9	1.9	0.9	1.7	0.8	2.9	1.5	2.7	1.5	2.3	1.2
Boys	2.1	1.0	2.0	0.9	1.8	0.8	3.3	1.5	2.9	1.6	2.5	1.2
Girls	1.9	0.9	1.9	0.9	1.6	0.8	2.5	1.4	2.6	1.3	2.2	1.3
Outdoor play time (hours/day)*	1.6	0.7	1.5	0.7	1.4	0.6	2.1	0.8	1.9	0.8	1.7	0.6
Have PC, TV, etc. in the room (%)*	43.7		35.8		22.6		51.6		43.1		26.5	
Outcome variables - food consumption frequencies (days/week)												
Legumes*	2.9	1.7	2.6	1.5	2.4	1.3	2.9	1.8	2.7	1.5	2.4	1.3
100% fruit juice	2.9	2.4	2.9	2.4	2.9	2.5	2.1	2.3	2.3	2.2	2.5	2.4
Whole-fat milk	3.9	3.2	3.9	3.2	3.7	3.3	3.3	3.2	3.4	3.2	3.4	3.3
Milkshakes*	2.3	2.3	2.0	2.2	1.7	2.2	2.2	2.1	2.1	2.2	1.7	2.0
Cheese*	2.8	2.2	3.0	2.2	3.1	2.2	2.5	2.0	2.8	2.1	3.0	2.1
Yoghourt, or other dairy products	4.7	2.2	4.7	2.2	4.7	2.2	4.7	2.2	4.6	2.3	4.7	2.3
Meat	3.5	1.9	3.6	1.8	3.8	1.8	3.5	1.9	3.5	1.9	3.8	1.8
Fish	2.6	1.6	2.6	1.4	2.7	1.5	2.7	1.6	2.6	1.5	2.8	1.5
Potato chips and other salty snacks*	2.0	1.3	1.9	1.3	1.6	1.2	2.1	1.3	1.9	1.1	1.7	1.1
Candy bars or chocolate	2.1	1.5	2.1	1.5	2.0	1.5	2.2	1.4	2.1	1.4	2.1	1.4
Biscuits, cakes, etc.	2.3	1.7	2.5	1.8	2.5	1.8	2.5	1.7	2.6	1.8	2.8	2.1
Pizza, chips, hamburgers	2.1	1.0	2.1	1.1	2.0	0.9	2.1	1.0	2.0	0.9	2.0	0.9
Eggs	2.2	1.3	2.4	1.4	2.3	1.3	2.1	1.0	2.3	1.1	2.3	1.2
Cereals	2.5	2.3	2.8	2.4	3.1	2.5	2.5	2.4	2.6	2.3	2.8	2.5
White bread	4.4	2.7	4.5	2.6	4.7	2.5	4.4	2.5	4.3	2.5	4.6	2.5
Whole-grain bread	0.8	1.7	0.8	1.8	0.9	1.8	0.7	1.7	0.8	1.7	1.0	1.8
Pasta	2.7	1.8	2.7	1.7	2.8	1.6	2.4	1.3	2.5	1.4	2.5	1.3
Fresh fruit*	3.9	2.4	4.5	2.4	5.3	2.2	4.1	2.4	4.6	2.3	5.3	2.2
Vegetables (excluding potatoes)*	2.8	1.9	3.2	2.0	3.8	2.1	2.8	2.0	3.5	2.1	4.0	2.1
Soft drinks containing sugar*	1.8	2.2	1.4	1.9	1.1	1.6	1.4	2.9	1.2	1.7	0.8	1.3
Diet/light soft drinks*	0.9	1.9	0.8	1.8	0.6	1.5	0.7	1.6	0.6	1.4	0.5	1.2

* *p* < 0.05 for mean differences between educational level groups

In 2011 and 2013, children who had parents with no formal or only primary school education, displayed a significantly higher consumption of “soft drinks containing sugar”, “diet/light soft drinks”, “legumes”, “milkshakes” and “potato chips and other salty snacks”, and a significantly lower consumption of “fresh fruit”, “vegetables (excluding potatoes)” and “cheese” (Table 3).

Mean outdoor playtime was 1.5 h in 2011 (1.5 in boys and 1.4 in girls, *p* < 0.05) and 1.8 h in 2013 (1.9 in boys and 1.7 in girls, *p* < 0.05). No differences were found in relation to body weight (Table 2).

In 2011, while 30.2% of children (32.1% of boys and 28.3% of girls) had a computer, television or games console in their bedrooms, there was a significant difference in this proportion between those who were overweight (34.7%) and those who had normal weight (26.7%). Moreover, this difference applied to boys and girls alike. In 2013, 35.1% of children (38.0% of boys and 32.3% of girls) had a computer, television or games console in their bedrooms, and a significant difference was again found between those who were overweight (38.4%) and those who had normal weight (32.6%); and, as in 2011, these differences applied to both sexes (Table 2). The proportion of children who had a computer, television or games console in their bedrooms was significantly higher among those whose parents had a lower level of education, in both 2011 and 2013. There were no significant differences in the proportion of boys and girls with a computer, television or games console in their bedrooms (Table 3).

Only minor differences in food consumption frequencies were observed between BMI categories, except for whole-fat milk, the consumption of which was significantly higher in the normal-weight group.

Some appreciable differences in food consumption frequencies were found between children with low versus high screen time, and between children with short versus long sleep duration (Table 4): “fresh fruit”, “vegetables (excluding potatoes)”, “100% fruit juice” (only in 2013), “cereals” (only in 2013), “fish” (only in 2013) and “whole-grain bread” (only in 2013) consumption frequencies were significantly higher in children with low screen time; and “fresh fruit”, “vegetables (excluding potatoes)”, “whole-fat milk”, “yoghurt, cream cheese or other dairy product” (only in 2011), “whole-grain bread” (only in 2013), “meat” (only in 2013) and “fish” (only in 2013) consumption frequencies were significantly higher in children with long sleep duration. The consumption frequencies of “potato chips and other salty snacks”, “candy bars or chocolate”, “biscuits, cakes, etc.”, “pizza, French fries (chips), hamburgers”, “milkshakes”, “soft drinks containing sugar” and “diet/light soft drinks” (only in 2013) were significantly higher in children with high screen time. The consumption frequencies of “soft drinks containing sugar”, “diet/light soft drinks” (only in 2011), “potato chips and other salty snacks” (only in 2011), “candy bars or chocolate” (only in 2013) and “milkshakes” (only in 2013) were significantly higher in children with short sleep duration. No statistically significant differences in food consumption frequencies were found between boys and girls.

**Table 4:** Mean food consumption frequencies (days/week) by screen time and sleep duration category

Food ítems	Screen time		Sleep duration	
	<2 h/day	≥2 h/day	<9.9 h/day	≥9.9 h/day
	Mean consumption frequencies in days per week (SD)			
2011				
Legumes	2.5 (1.4)	2.6 (1.6)	2.5 (1.5)	2.6 (1.4)
100% fruit juice	2.9 (2.5)	2.8 (2.5)	2.8 (2.5)	2.9 (2.5)
Whole-fat milk	3.8 (3.3)	3.9 (3.2)	**3.6** (3.2)	**3.9** (3.3)
Milkshakes	**1.7** (2.1)	**2.2** (2.3)	1.9 (2.2)	1.8 (2.2)
Cheese	3.1 (2.2)	3.0 (2.2)	3.0 (2.2)	3.1 (2.2)
Yoghurt, or other dairy products	4.7 (2.2)	4.8 (2.2)	**4.6** (2.2)	**4.8** (2.2)
Meat	3.7 (1.8)	3.7 (1.9)	3.6 (1.9)	3.7 (1.8)
Fish	2.7 (1.5)	2.6 (1.4)	2.6 (1.5)	2.7 (1.5)
Potato chips and other salty snacks	**1.6** (1.2)	**2.1** (1.4)	**1.9** (1.3)	**1.7** (1.2)
Candy bars or chocolate	**1.9** (1.4)	**2.2** (1.6)	2.1 (1.5)	2.0 (1.5)
Biscuits, cakes, etc.	**2.3** (1.7)	**2.7** (1.9)	2.5 (1.8)	2.4 (1.8)
Pizza, chips, hamburgers	**2.0** (1.0)	**2.2** (1.1)	2.1 (1.1)	2.0 (1.0)
Eggs	2.3 (1.3)	2.3 (1.3)	2.3 (1.3)	2.3 (1.3)
Cereals	2.9 (2.5)	2.8 (2.4)	2.8 (2.5)	3.0 (2.5)
White bread	4.6 (2.5)	4.5 (2.6)	4.5 (2.6)	4.6 (2.5)
Whole-grain bread	0.9 (1.8)	0.9 (1.8)	0.9 (1.8)	0.9 (1.7)
Pasta	2.7 (1.6)	2.8 (1.7)	2.8 (1.7)	2.7 (1.6)
Fresh fruit	**5.1** (2.2)	**4.3** (2.4)	**4.6** (2.4)	**5.0** (2.3)
Vegetables (excluding potatoes)	**3.6** (2.1)	**3.2** (2.0)	**3.3** (2.0)	**3.6** (2.0)
Soft drinks containing sugar	**1.1** (1.6)	**1.6** (2.0)	**1.4** (1.9)	**1.2** (1.7)
Diet/light soft drinks	**0.6** (1.5)	**0.9** (1.9)	**0.8** (1.8)	**0.6** (1.5)
2013				
Legumes	2.5 (1.3)	2.6 (1.5)	2.6 (1.5)	2.6 (1.4)
100% fruit juice	**2.6** (2.4)	**2.3** (2.3)	2.3 (2.3)	2.5 (2.3)
Whole-fat milk	3.4 (3.3)	3.3 (3.3)	**3.1** (3.2)	**3.5** (3.3)
Milkshakes	**1.6** (2.0)	**2.0** (2.2)	**2.0** (2.2)	**1.7** (2.0)
Cheese	2.9 (2.1)	2.8 (2.1)	2.9 (2.1)	2.9 (2.1)
Yoghurt, or other dairy products	4.6 (2.2)	4.7 (2.2)	4.6 (2.3)	4.7 (2.2)
Meat	3.7 (1.8)	3.6 (1.9)	**3.5** (1.9)	**3.7** (1.8)
Fish	**2.9** (1.6)	**2.6** (1.4)	**2.6** (1.5)	**2.8** (1.5)
Potato chips and other salty snacks	**1.6** (1.0)	**2.0** (1.2)	1.9 (1.2)	1.8 (1.1)
Candy bars or chocolate	**1.9** (1.2)	**2.2** (1.5)	**2.2** (1.5)	**2.0** (1.3)
Biscuits, cakes, etc.	**2.6** (1.9)	**2.8** (2.0)	2.8 (2.0)	2.7 (2.0)
Pizza, chips, hamburgers	**1.9** (0.8)	**2.1** (1.0)	2.1 (1.0)	2.0 (0.9)
Eggs	2.3 (1.1)	2.3 (1.2)	2.3 (1.1)	2.3 (1.1)
Cereals	**2.9** (2.5)	**2.6** (2.4)	2.7 (2.5)	2.7 (2.4)
White bread	4.5 (2.5)	4.5 (2.5)	4.4 (2.5)	4.5 (2.5)
Whole-grain bread	**1.0** (1.9)	**0.8** (1.7)	**0.8** (1.7)	**1.0** (1.8)
Pasta	2.4 (1.2)	2.5 (1.4)	2.5 (1.4)	2.4 (1.3)
Fresh fruit	**5.3** (2.2)	**4.7** (2.3)	**4.6** (2.3)	**5.2** (2.2)
Vegetables (excluding potatoes)	**3.9** (2.1)	**3.6** (2.1)	**3.5** (2.1)	**3.8** (2.1)
Soft drinks containing sugar	**0.7** (1.3)	**1.2** (1.7)	**1.1** (1.6)	**0.9** (1.4)
Diet/light soft drinks	0.5 (1.2)	0.6 (1.3)	0.6 (1.4)	0.5 (1.3)

Bold figures indicate statistically significant mean difference (*p* < 0.05) between both categories

No relevant differences in exposure variables and food consumption frequencies were found between age groups.

The adjusted odds ratios (ORs) and 95% confidence intervals (CIs) from the multivariable analyses are shown in Table 5. One additional hour of screen time was associated with a significantly higher consumption of: “soft drinks containing sugar”, “diet/light soft drinks”, “milkshakes”, “potato chips and other salty snacks”, “candy bars or chocolate”, “biscuits, cakes, etc.”, “pizza, French fries (chips), hamburgers” and “legumes” in 2011 and 2013; and “eggs” and “pasta” in 2013.

**Table 5:** Adjusted odds ratio ^a^ and 95%CI for the effects of screen time (hours/day) and total sleep duration (hours/day) on consumption frequencies of selected food items among children aged 6 to 9 years: 2011 and 2013

	Screen time (hours/day)		Sleep duration (hours/day)	
	OR	95%CI	OR	95%CI
2011				
Legumes	1.09	(1.01; 1.18)*	0.98	(0.90; 1.08)
100% fruit juice	0.96	(0.93; 1.05)	1.10	(1.02; 1.20)*
Whole-fat milk	1.03	(0.98; 1.10)	1.17	(1.09; 1.26)**
Milkshakes	1.20	(1.11; 1.29)**	0.94	(0.85; 1.03)
Cheese	1.01	(0.95; 1.08)	1.04	(0.97; 1.13)
Yoghurt, or other dairy products	1.00	(0.95; 1.08)	1.06	(0.98; 1.14)
Meat	1.04	(0.98; 1.10)	1.02	(0.95; 1.10)
Fish	0.85	(0.79; 0.92)**	1.10	(1.01; 1.21)*
Potato chips and other salty snacks	1.64	(1.47; 1.83)**	0.88	(0.76; 1.03)
Candy bars or chocolate	1.40	(1.28; 1.53)**	0.87	(0.80; 0.98)*
Biscuits, cakes, etc.	1.31	(1.22; 1.41)**	0.94	(0.90; 1.04)
Pizza, chips, hamburgers	1.37	(1.22; 1.54)**	0.84	(0.72; 0.99)*
Eggs	1.03	(0.94; 1.13)	0.98	(0.87; 1.09)
Cereals	0.98	(0.92; 1.05)	1.14	(1.06; 1.23)*
White bread	0.99	(0.93; 1.05)	1.03	(0.96; 1.11)
Whole-grain bread	0.99	(0.88; 1.11)	0.98	(0.86; 1.12)
Pasta	1.00	(0.93; 1.08)	0.88	(0.80; 0.96)*
Fresh fruit	0.75	(0.70; 0.80)**	1.19	(1.10; 1.28)**
Vegetables (excluding potatoes)	0.78	(0.71; 0.86)**	1.18	(1.06; 1.31)*
Soft drinks containing sugar	1.33	(1.25; 1.42)**	0.94	(0.87; 1.01)
Diet/light soft drinks	1.14	(1.06; 1.22)*	0.92	(0.83; 0.99)*
2013				
Legumes	1.08	(1.00; 1.15)*	1.02	(0.89; 1.18)
100% fruit juice	0.91	(0.85; 0.98)*	0.96	(0.84; 1.09)
Whole-fat milk	0.98	(0.92; 1.04)	1.15	(1.02; 1.29)*
Milkshakes	1.11	(1.03; 1.20)*	0.84	(0.73; 0.98)*
Cheese	1.03	(0.97; 1.10)	0.99	(0.88; 1.12)
Yoghurt or other dairy products	1.00	(0.94; 1.06)	1.14	(1.02; 1.28)*
Meat	1.03	(0.98; 1.10)	1.20	(1.07; 1.34)*
Fish	0.87	(0.81; 0.94)*	1.14	(0.99; 1.30)
Potato chips and other salty snacks	1.45	(1.29; 1.62)**	0.91	(0.71; 1.19)
Candy bars or chocolate	1.36	(1.25; 1.48)**	0.85	(0.71; 1.02)
Biscuits, cakes, etc.	1.12	(1.05; 1.20)*	0.95	(0.83; 1.09)
Pizza, chips, hamburgers	1.38	(1.23; 1.55)**	0.90	(0.69; 1.16)
Eggs	1.14	(1.04; 1.24)*	0.92	(0.77; 1.09)
Cereals	0.97	(0.91; 1.03)	0.99	(0.88; 1.12)
White bread	0.99	(0.93; 1.05)	1.02	(0.91; 1.15)
Whole-grain bread	0.85	(0.76; 0.96)*	1.23	(0.99; 1.52)
Pasta	1.15	(1.07; 1.24)**	0.86	(0.74; 0.99)*
Fresh fruit	0.80.	(0.75; 0.85)**	1.35	(1.20; 1.52)**
Vegetables (excluding potatoes)	0.85	(0.78; 0.92)**	1.22	(1.05; 1.42)*
Soft drinks containing sugar	1.23	(1.21; 1.37)**	0.99	(0.88; 1.11)
Diet/light soft drinks	1.13	(1.06; 1.22)*	0.92	(0.80; 1.06)

CI confidence interval, OR odds ratio

Significance levels: * *p* < 0.05; ** *p* < 0.001

^a^ All models were adjusted for sex, age, outdoor playtime, maximum parental level of education and body mass index category

One additional hour of screen time was also associated with a significantly lower consumption of: “fresh fruit”, “vegetables (excluding potatoes)” and “fish” in 2011 and 2013: and “100% fruit juice” and “whole-grain bread” in 2013.

One additional hour of sleep was associated with: a significantly higher consumption of “fresh fruit”, “vegetables (excluding potatoes)” and “whole-fat milk” in 2011 and 2013; and a higher consumption of “100% fruit juice”, “fish” and “cereals” in 2011 and “yoghurt, cream cheese or other dairy products”, and “meat” in 2013. Conversely, one additional hour of sleep was significantly associated with a lower consumption of “pasta” in 2011 and 2013, “candy bars or chocolate”, “pizza, French fries (chips), hamburgers” and “diet/light soft drinks” in 2011, and “milkshakes” in 2013.

## Discussion

There has been extensive study into the relationship between time devoted to screen-based forms of entertainment (such as television, video games, and especially in recent years, computers, telephones and tablets), hours of sleep and the presence of overweight and obesity [9, 12, 25–35]. In contrast, there have been far fewer studies that have examined the relationship between screen time and sleep time on the one hand, and dietary habits and the consumption of different foods on the other [10, 36, 37]. This study presents evidence to show that both sleep duration and screen time are related to the consumption of different foods by children of both sexes aged 6 to 9 years, and that this relationship is constant, as it is repeated over time.

In general, screen time was associated with a higher frequency of consumption of energy-dense, micronutrient-poor products, with the single exception of legumes, which are a basic component of the Mediterranean diet. This association is consistent with the literature [25, 38, 39], and it is therefore important to propose setting limits on children’s exposure to screen-based forms of entertainment [40].

One explanation for these findings may be that passive consumption of products rich in fats, salt and sugar is higher among children who spend more time in front of screens because such children tend to pay less attention to what they eat when simultaneously engaging in some other activity [10, 31]. Furthermore, many of the television advertisements scheduled during children’s programming are for foods and drinks, many of which have a high energy, salt, fat and/or sugar content [41, 42]. In addition, children are exposed to advertisements for foods and drinks on the Internet [43].

Exposure to advertising is also a possible explanation for overconsumption of foods and drinks for which food guides advise occasional consumption [44].

The low consumption of fruit and vegetable associated with higher amounts of screen time may be caused by a substitution effect, due to competition with the wider range of and advertisements for high-energy products [45]. This is despite the fact that greater exposure to fruit and vegetable advertisements has been shown to be associated with a higher consumption of such products [46].

Analysis of the other exposure variable revealed that one additional hour of sleep was associated with a significantly higher consumption of, among other things, “fresh fruit”, “vegetables (excluding potatoes)” and “whole-fat milk” in 2011 and 2013.

There are several mechanisms that may account for this. Firstly, it is possible that, due to sleeping more, children may have less time in which to eat [47]. Additionally, by sleeping more, they watch less television and are less exposed to advertising. Another hypothesis is that there may be neurohormonal changes which cause reduced levels of sleep to produce greater appetite and calorie intake [12, 15, 48]. The cross-sectional nature of this study means that the precise causes of this relationship, i.e., between screen time, sleep duration and eating patterns, cannot be established with certainty. Hence, additional studies would be required to evaluate this.

It is also important to take into account the fact that the findings demonstrate significant differences in the consumption of certain foods, depending on parents’ level of education, inasmuch as the lower their level of education, the higher the consumption of sugar- and fat-rich foods and the lower the consumption of fruit and vegetables. In addition, lower levels of education were found to be associated with a greater proportion of children with passive forms of entertainment in their bedrooms and children with overweight. This suggests that, in addition to establishing overarching interventions, the social strata in which lower levels of education may be found should be the subject of targeted strategies and interventions. Although our study assessed the level of education of mothers and fathers separately, there were no consistent differences and thus no consistent results. It is possible that a larger sample size might reveal a different association between mothers’ and fathers’ respective educational levels and the frequency of consumption of certain foods.

Nutritional status is related to sleep time, screen time and the consumption of different nutrients; however, the mechanisms through which all these variables interact are unclear. To avoid a potential confounding effect caused by nutritional status, the regression models were adjusted for this variable.

There was a significant increase in screen time in 2013 compared to 2011, though outdoor playtime also increased over the same period. In order to monitor this increase, average screen time would have to be assessed by future studies.

### Strengths and limitations

Our study has several strengths, one of which is its large sample size, with almost 2000 children of each age, which allows for more accurate estimates. Furthermore, it was conducted using a methodology that was standardised according to the COSI protocols used in many countries. The concordance of the findings of the 2011 and 2013 surveys is striking, and serves to reinforce the study’s strength. The external validity of these results is guaranteed by the sampling design and large sample size.

The main limitation lies in the fact that the ALADINO Study has a cross-sectional design, meaning that, while it may point to associations, causation cannot be established. Another limitation may be that estimated sleep duration and screen time were reported by parents and may thus not be completely accurate. However, as the sample was sufficiently large, this error could be assumed to be randomly distributed and so not bias the estimated relationships between variables.

## Conclusions

High screen time and short sleep duration were associated with increased BMI, higher frequencies of consumption of energy-dense, micronutrient-poor products (such as sugar-sweetened soft drinks, snacks and chocolates), and lower frequencies of consumption of fruit and vegetables. Mean screen time was significantly higher among the children of parents with no formal or only primary school education than among those of parents with a university education.

Education continues to be one of the key elements for improving diet. It is for this reason that action must be taken to communicate and raise awareness about the importance to children of a balanced diet, regular physical activity, recommended number of hours of sleep and limited exposure to passive forms of entertainment, such as television, video games, mobile phones and computers. Moreover, actions of this nature should be particularly targeted at families having a lower level of education.

## Abbreviations

ALADINO: *Alimentación, Actividad Física*, *Desarrollo Infantil y Obesidad* (Nutrition, Physical Activity, Childhood Growth and Obesity)
BMI: Body mass index
COSI: Childhood Obesity Surveillance Initiative
FFQ: Food frequency questionnaire
WHO: World Health Organisation
